# SARS-CoV-2 specific sIgA in saliva increases after disease-related video stimulation

**DOI:** 10.1038/s41598-023-47798-y

**Published:** 2023-12-20

**Authors:** Judith K. Keller, Alex Dulovic, Jens Gruber, Johanna Griesbaum, Nicole Schneiderhan-Marra, Clemens Wülfing, Jana Kruse, Annika Hartmann, Esther K. Diekhof

**Affiliations:** 1 Department of Biology, Neuroendocrinology and Human Biology Unit, Faculty of Mathematics, Informatics and Natural Sciences, Institute for Animal Cell and Systems Biology, Universität Hamburg, Martin-Luther-King-Platz 3, 20146 Hamburg, Germany; 2 NMI Natural and Medical Sciences Institute at the University of Tübingen, Reutlingen, Germany; 3 Department of Biology, Interdisciplinary Neurobiology and Immunology, Faculty of Mathematics, Informatics and Natural Sciences, Institute for Animal Cell and Systems Biology, Universität Hamburg, Hamburg, Germany

**Keywords:** Human behaviour, Mucosal immunology

## Abstract

Secretory immunoglobulin A (sIgA) in saliva is the most important immunoglobulin fighting pathogens in the respiratory tract and may thus play a role in preventing SARS-CoV-2 infections. To gain a better understanding of the plasticity in the mucosal antibody, we investigated the proactive change in secretion of salivary SARS-CoV-2-specific sIgA in 45 vaccinated and/or previously infected, generally healthy persons (18 to 35 years, 22 women). Participants were exposed to a disease video displaying humans with several respiratory symptoms typical for COVID-19 in realistic situations of increased contagion risk. The disease video triggered an increase in spike-specific sIgA, which was absent after a similar control video with healthy people. The increase further correlated inversely with revulsion and aversive feelings while watching sick people. In contrast, the receptor binding domain-specific sIgA did not increase after the disease video. This may indicate differential roles of the two salivary antibodies in response to predictors of airborne contagion. The observed plasticity of spike-specific salivary antibody release after visual simulation of enhanced contagion risk suggests a role in immune exclusion.

## Introduction

Since the initial outbreak of SARS-CoV-2 in Wuhan, China, in late 2019^1^, COVID-19 evolved rapidly into a global pandemic, in part due to its airborne transmissibility that even further increased with emerging variants of concern. Its primary route of transmission through respiratory droplets and aerosols^2^ suggests that the mucosal immune response in the oral and nasal cavities may be important for limiting viral infection. Within this context, secretory immunoglobulin A (sIgA) in saliva could play a significant role in preventing SARS-CoV-2 infections, as this is the most important immunoglobulin fighting pathogens in the respiratory tract^3^. SIgA is secreted by plasma cells adjacent to the mucosal epithelial cells^4^. It binds antigens and prevents their attachment to epithelial cells and is further involved in intracellular neutralization of viral replication, thus significantly contributing to immune exclusion^4^. Given these functions, sIgA may also have the potential of neutralizing SARS-CoV-2^3^. In fact, during the early stages of a SARS-CoV-2 infection SARS-CoV-2-specific IgA does not only dominate the humoral immune responses in serum, bronchoalveolar fluid and saliva, but it was also found to be more strongly correlated with the neutralization of the virus than the immunoglobulins M and G^5^. Furthermore, higher sIgA in saliva and nasal mucus has been associated with asymptomatic as opposed to symptomatic COVID-19-infections, which might also hint at its protective role against SARS-CoV-2^6,7^. Recent research findings further observed an increase in SARS-CoV-2-specific antibodies in saliva following intramuscular vaccination with the approved messenger ribonucleic acid (mRNA) vaccines developed by Pfizer/BioNTech (BNT-162b2) and Moderna (mRNA-1273)^8–10^. Also, the sIgA titer after vaccination seemed to be somewhat lower in people, who have not been previously infected with SARS-CoV-2^9,11^. Therefore, it would be interesting to know if the body has additional ways to transiently enhance the mucosal antibody level after vaccination, especially required in certain situations with heightened contagion risk that cannot be easily avoided.

For other viruses (e.g., influenza viruses), it has already been shown that the virus-specific antibody level in saliva can be enhanced on demand^12^, if a person had already acquired the respective antibody repertoire through previous vaccination or infection. It thus seems plausible that following initial contact with COVID-19, either through infection or vaccination, the organism should be able to increase the release of SARS-CoV-2-specific sIgA in saliva whenever needed (e.g., after viral exposure). Interestingly, a number of psychoimmunological studies have recently demonstrated that actual pathogen exposure is not always obligatory to trigger a mucosal immune response. In fact, several immune markers in saliva and serum were found to respond proactively to the mere expectation of pathogen exposure, by showing an increase following visual stimulation with general disease-related content^13–16^. This was also the case for total sIgA in saliva, which increased after a video of people exhibiting typical symptoms of respiratory diseases (e.g., sneezing and coughing)^17^. Collectively, these findings led us to hypothesize that visual disease predictors, such as a video displaying people with respiratory symptoms, should trigger a proactive release of SARS-CoV-2-specific sIgA in a similar way in vaccinated individuals, and might thus transiently increase mucosal immunity temporarily even in the absence of the actual coronavirus. Such a proactive and virus-specific increase would be adaptive, given the high number of infected people in the population and the permanent risk of viral exposure.

**Figure 1:**
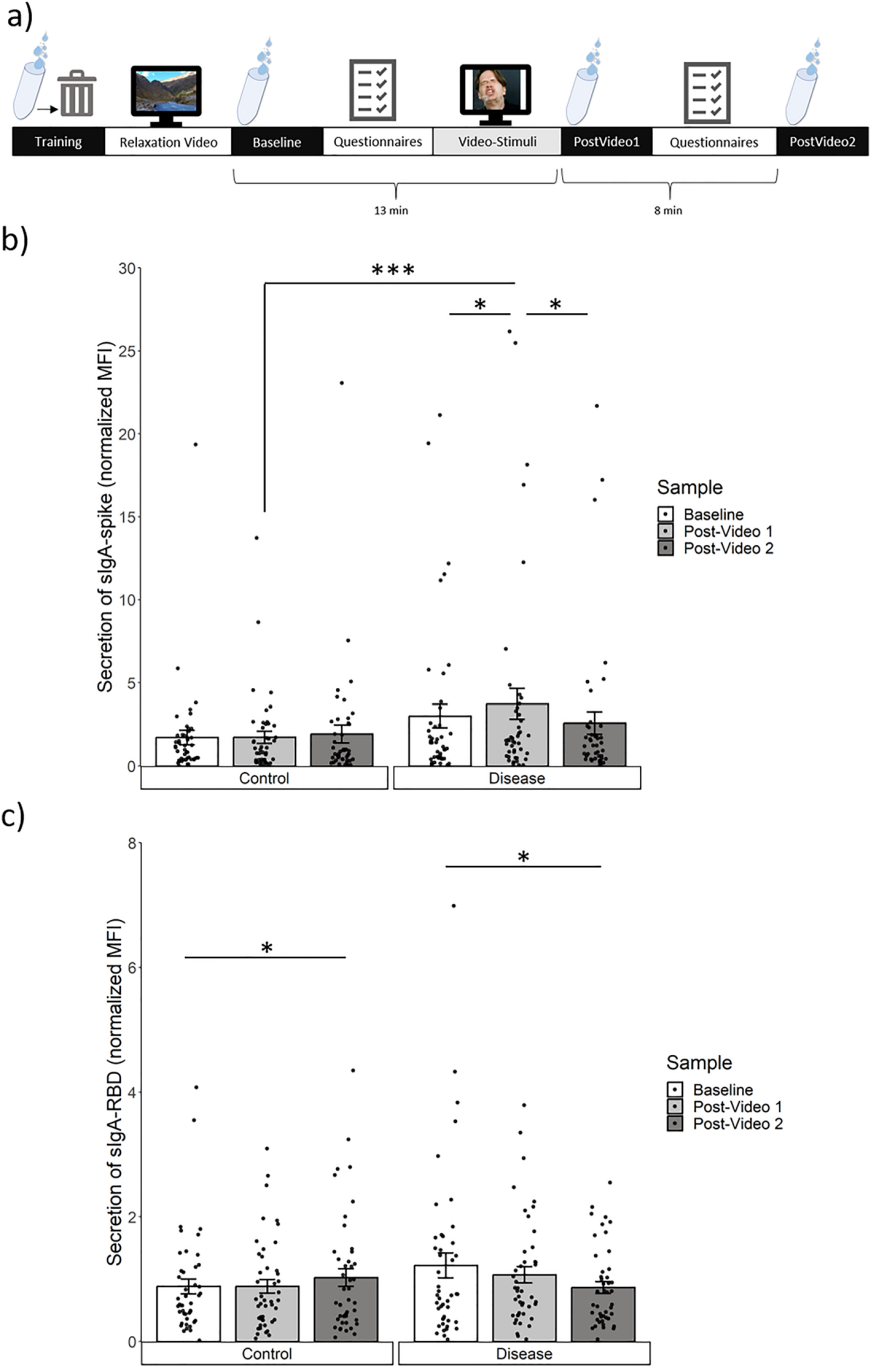
Antibody changes across test procedure. (**A**) Schematic of the test procedure: Temporal order of relaxation video and questionnaires (white), saliva samples (black), and stimulation video (gray) on each test day. Average time (in min) between the starting points of the saliva samples is indicated below the chart. (**B**) Spike-specific sIgA: Bar plot with mean, standard errors, and individual data points of the secretion rate at Baseline, directly after the video (Post-Video 1), and several minutes after the video (Post-Video 2). (**C**) RBD-specific sIgA: Bar plot with mean, standard errors, and individual data points of the secretion rate at Baseline, Post-Video 1, and Post-Video 2. Significant changes are marked with asterisks (*p < .05; ***p < .001), based on Wilcoxon signed rank test.

To evaluate this we utilized an adapted test protocol from the study by Keller et al.^17^. The design comprised two within-subject test sessions, during which we measured SARS-CoV-2-specific sIgA and collected self-report state-measures of disgust and interoceptive feelings following a standardized test protocol (Fig. 1a). On two separate days, the participants either watched a disease video displaying people with respiratory symptoms or a control video with healthy people. Before and after the video, we measured SARS-CoV-2-specific sIgA to the spike and receptor-binding domain (RBD) antigens in saliva in order to assess their change from baseline to after the video. Based on our previous findings of a proactive increase in the total salivary sIgA following visual exposure with disease-related content^17^, we expected the SARS-CoV-2-specific sIgA secretion to increase after the disease video displaying people with respiratory symptoms, but not after the control video.

In addition to that, our participants completed the *perceived Vulnerability to Disease Questionnaire*^18^ and the revised *Disgust Scale*^19^ some days before the first test took place. Further, state disgust and interoceptive feelings in response to the video were assessed during each test session according to the predetermined schedule^20,21^. It has previously been shown that both an increased disgust propensity and the acute feeling of revulsion may reduce contagion risk by proactively triggering the behavioral avoidance of an increased pathogen threat, which should in turn reduce the need for an enhanced physiological immune response^17,18,22^. Based on this evidence, we expected the different trait and state measures of disgust, disease propensity, and interoceptive feelings to negatively correlate with a proactive increase in SARS-CoV-2-specific sIgA in response to the disease video.

## Results

We excluded the data of one participant, who was an outlier in all spike-specific and three of the RBD-specific sIgA samples (SARS-CoV-2-specific sIgA > two standard deviations above sIgA mean). The cohort used in this study was evenly male (n = 23) and female (n = 22), with an average age of 25.4 years (σ = 4.39). All participants were vaccinated against SARS-CoV-2, which was also indicated by their average IgG-titer in blood (mean = 993.33 BAU/mL; σ = 153.7) (see SI Table S1).

### SARS-CoV-2-specific sIgA increase after disease-related stimulation

#### Spike-specific sIgA

In order to assess whether the disease video led to an increase in spike-specific sIgA, we performed a generalized linear mixed model (GLMM) with the factors *Video* (disease, control) and *Sample* (Baseline, Post-Video 1, Post-Video 2) as well as the random factor *Subject* with a gamma-log linked distribution as sIgA-data was left skewed. We found a significant main effect of *Video* (F_(1,264)_ = 20.64, *p* =  < .001) and *Sample* (F_(2,264)_ = 3.83, *p* = .023) as well as a significant interaction between the two factors (F_(2,264)_ = 6.16, *p* = .002, find fixed coefficients in SI Table S5). In the post-hoc tests, this was reflected by a significant rise in spike-specific sIgA in the sample directly collected after watching the disease video (Post-Video 1) relative to Baseline (z = − 1.80, *p* = .036, η^2^ = .72), but not in the corresponding sample taken after the control video (z = − .46, *p* = .648). Additionally, spike-specific sIgA significantly declined from Post-Video 1 to Post-Video 2 after watching the disease video (z = − 2.56, *p* = .011, η^2^ = .15), but not after the control video (z = − .12, *p* = .906). Finally, we found that the samples collected at Post-Video 1 differed significantly between the two videos (disease > control: z = − 3.22, *p* < .001, η^2^ = .23), while the Baseline (z = − 1.59, *p* = .113) and Post-Video 2 samples (z = − 1.25, *p* = .212) did not (Fig. 1b).

We further ran an explorative analysis of *Video Order* as a covariate in the model, since it cannot be ruled out that the first video the participants had watched may have had an influence on sIgA secretion on the second test day. For full analysis see SI Results 2.4. In this analysis, we found a significant 3-way interaction between Video*Sample*Video Order (F_(2,258)_ = 7.33, *p* < .001). When data was split according to *Video Order*, post-hoc tests on ΔsIgA showed that the increase between Baseline and Post-Video 1 was only significantly higher after the disease video compared to control video, when participants saw the disease video first (z = − 2.71, *p* = .007, η^2^ = .16), but not when they saw the control video first (z = 1.43, *p* = .153).

Finally, we ran a confirmatory analysis of total sIgA, which had significantly increased in response to disease-related video content in our previous study^17^.We found that total sIgA showed a similar response to the present disease video as spike-specific sIgA in that it showed a stronger increase after the disease than following the control video (z = − 1.75, *p* = .040, η^2^ = .07) (see SI Results 2.3). The ΔsIgA_total_ was further positively correlated with ΔsIgA_spike_ for the disease video (rho = .593, *p* < .001).

#### RBD-specific sIgA

In a second step, we analyzed the RBD-specific sIgA for changes induced by the disease video. In the GLMM we neither found a significant main effect of *Video* (F_(1,264)_ = 3.10, *p* = .079) nor of *Sample* (F_(2,264)_ = .82, *p* = .444), but there was a significant interaction between the two factors (F_(2,264)_ = 6.79, *p* = .001, find fixed coefficients in SI Table S6). Different from the spike-specific sIgA, the RBD-specific sIgA showed no significant rise from Baseline to directly after the disease video (ΔsIgA_RBD_: z = − .37, *p* = .714), and - similar to the spike-specific sIgA - also not after the control video (ΔsIgA_RBD_: z = − .04, *p* = .968). Instead, we found a trend-wise decline in the RBD-specific sIgA from Post-Video 1 to Post-Video 2 (z = − 1.95, *p* = .052, η^2^ = .08), and also from Baseline to Post-Video 2 (z = − 2.18, *p* = .029, η^2^ = .11) following the disease video. This indicated a continuous decrease in RBD-specific sIgA throughout the experimental session with the disease video. After the control video, we found a significant increase between Post-Video 1 and Post-Video 2 (z = − 2.07, *p* = .038, η^2^ = .10), and also when comparing Baseline and Post-Video 2 (z = − 2.18, *p* = .029, η^2^ = .11) (see Fig. 1c).

### Trait disgust and perceived vulnerability to disease

The proactive increase in spike-specific sIgA in response to the disease video (ΔsIgA_spike_) neither correlated with the *Disgust Scale* (rho = .083, *p* = .294) nor with its subscales *Core Disgust* (rho = .005, *p* = .487) and *Contamination Disgust* (rho = .193, *p* = .102). Similarly, we found no significant relationship between ΔsIgA_spike_ and the total score of *perceived Vulnerability to Disease* (rho = − .172, *p* = .129) and also not with its subscales *Germ Aversion* (rho = − .108, *p* = .239) and *Perceived Infectability* (rho = − .190, *p* = .105).

### State interoceptive and emotional reactions to the disease video

**Figure 2:**
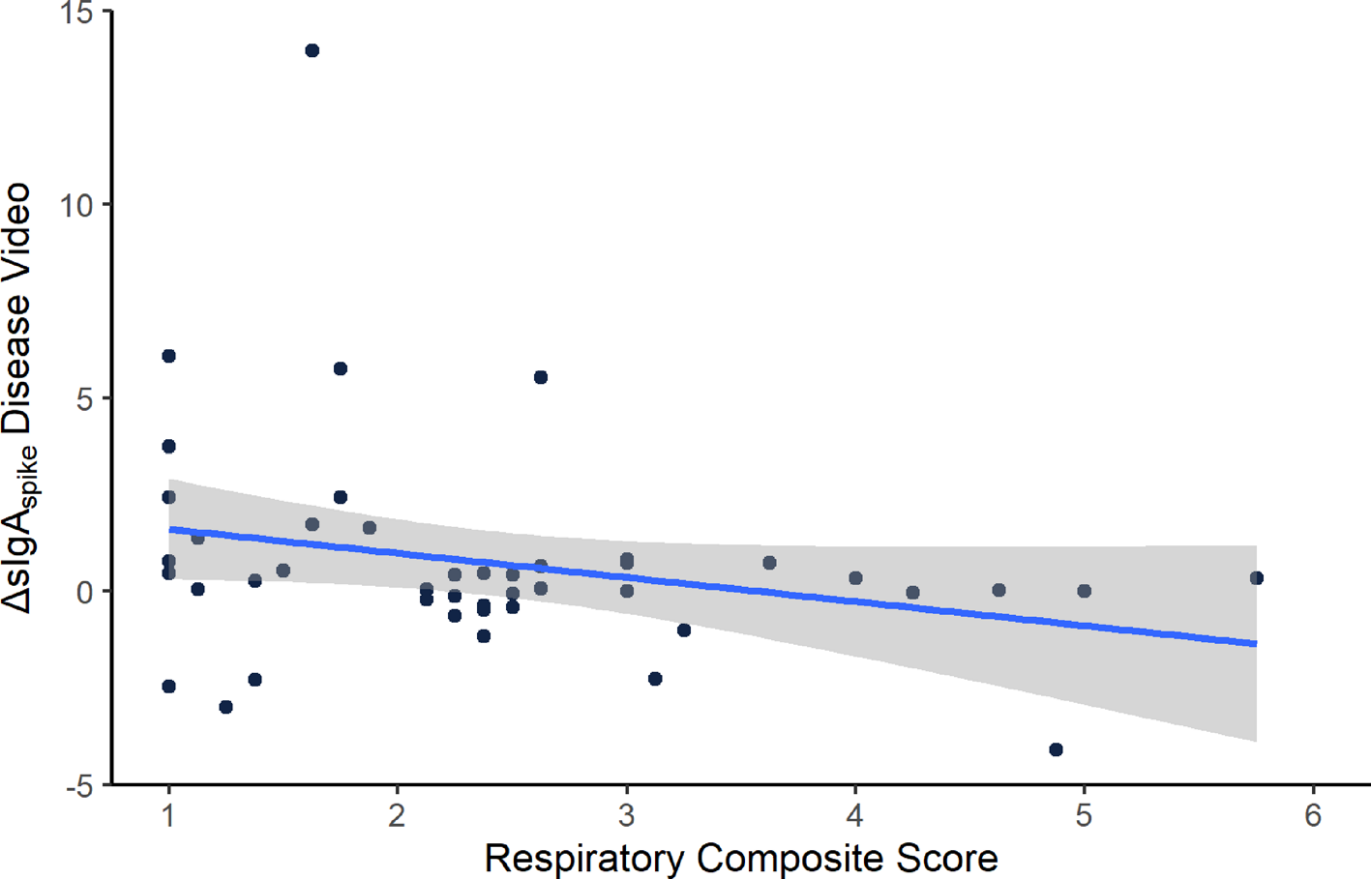
Interoceptive feelings in relation to spike-specific sIgA increase. Inverse correlation (*rho=-.299, p* =  .023) between ΔsIgA_spike_ after the disease video and interoceptive feelings as measured by the *Respiratory Composite Score* (i.e., the combined score of items related to oral, contamination-associated and flu-like interoceptive feelings; see SI Table S4). Scatter plot with a linear model based on the data with 95% confidence interval in gray.

After having watched the given video, participants answered self-report questions on their feelings experienced during the disease video. We found that ΔsIgA_spike_ correlated inversely with the adapted *Respiratory Composite Score* (rho = − .299, *p* = .023, Fig. 2) of the *Interoceptive Feelings Questionnaire* (see also SI Table S4). The subscale of *Feelings in the Gut* only showed a trend-wise negative correlation with ΔsIgA_spike_ (rho = − .212, *p* = .081).

**Figure 3:**
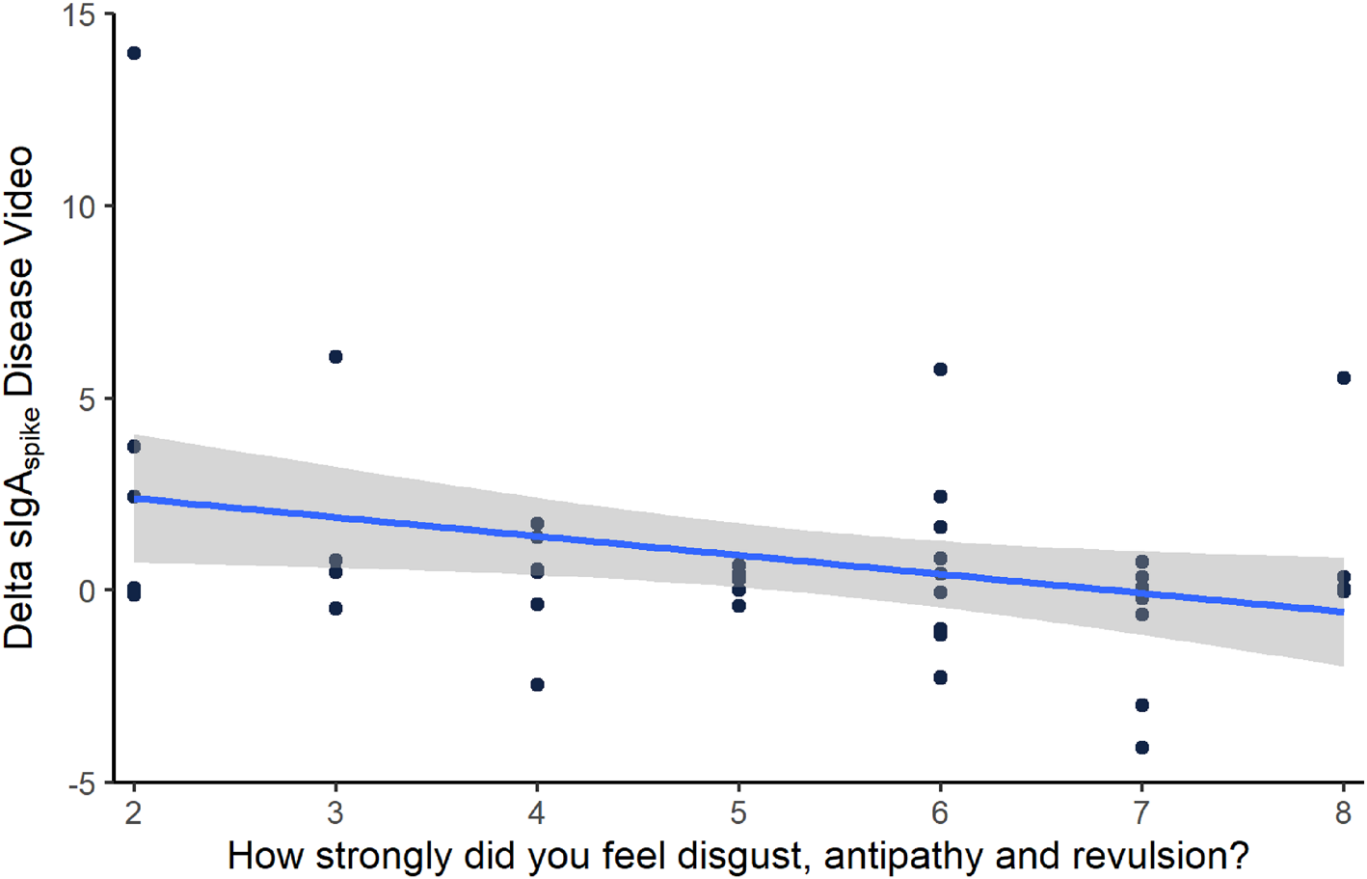
State disgust in relation to spike-specific sIgA increase. Inverse correlation (*rho=-.268, p* = .037) between ΔsIgA_spike_ and state disgust experienced during the disease video (Question: “*Please describe your emotions during the video: How strongly did you feel disgust, antipathy and revulsion?*”; 8 point-likert scale: 1 = “*I didn’t feel like this at all*” to 8 = “*I felt completely like that*”). Scatter plot with linear model based on the with 95% confidence interval in grey.

Additionally, the more state disgust participants indicated in the question “*How strongly did you feel disgust, antipathy and revulsion?*” after the disease video, the lower was their ΔsIgA_spike_ (rho = − .268, *p* = .037) (Fig. 3). In contrast, the average disgust rating of the screenshots from the disease video was not significantly correlated with ΔsIgA_spike_ (rho = − .159, *p* = .149).

## Discussion

SIgA in saliva is an important part of the first line of defense against respiratory diseases such as COVID-19. So far, research on SARS-CoV-2-specific sIgA mainly focused on antibody titers in serum and saliva following vaccination, infection or passive transfer^23–25^. This study investigated the proactive change in secretion of SARS-CoV-2-specific sIgA to a video displaying people with respiratory symptoms typical for COVID-19. By this, we wanted to achieve a better understanding of the plasticity in the antibody response to situations with heightened contagion potential. We found the predicted increase in the spike-specific sIgA after the disease video, but not following a video with healthy people. The increase in spike-specific sIgA closely resembled the increase in total sIgA as shown in the confirmatory analysis and in line with our previous results^17^. This suggests that this SARS-CoV-2-specific component of sIgA may serve a similar proactive function in immune exclusion as previously described for total sIgA^4,17^. The ΔsIgA_spike_ further correlated inversely with state disgust and feelings of discomfort in the oral cavity and respiratory tract, suggesting a compensatory relationship between psychological and physiological defensive reactions to predictors of airborne contagion. In contrast, the RBD-specific sIgA did not increase after the disease video, but declined from Baseline to Post-Video 2, which may indicate rather differential roles of the two specific salivary antibodies in response to predictors of airborne contagion.

Antibodies against the spike protein of SARS-CoV-2 are particularly important as the spike protein includes the RBD, which is the main target of neutralizing antibodies^26^. Both spike- and RBD-specific IgA are not only found in serum of vaccinated or previously infected persons, but both antibodies occur in meaningful amounts in saliva as well^8–11^. We^17^ and others^13^ have previously demonstrated a transient, proactive increase in total sIgA initiated by visual cues of increased contagion risk. Such a quick rise in sIgA is possible as sIgA is constantly secreted into saliva even at baseline and can be rapidly upregulated by (para-)sympathetic^27^ and mechanical stimulation^28^. The present observation of a significant rise in spike-specific sIgA by a median of 27.90% (Q_1_: 17.33%, Q_3_: 150.24%) following the ~ 8 min of mere visual experience of sneezing, coughing or otherwise sick persons, as well as its return back to baseline value shortly after the end of visual stimulation is consistent with these previous findings. The fact that this increase occurred in the absence of actual pathogen exposure indicates that the spike-specific sIgA could be part of a proactive immunological response that prepares the oral cavity for viral entry. We would therefore suggest that—similar to total sIgA– the spike-specific sIgA may be involved in immune exclusion rather than the actual neutralization of SARS-CoV-2^4^. This function would be quite adaptive, as heightened wild-type spike-specific sIgA in the mucosa has been observed to decrease the risk of infection even by the more contagious Omicron variant^29^. Apart from that, our data also showed that the RBD-specific sIgA did not follow the hypothesized pattern of a rise after the respiratory disease video, but - different from the spike-specific sIgA - declined over the course of the experiment. In contrast to anti-spike, RBD-specific antibodies have been shown to play a major role in neutralizing SARS-CoV-2^30–32^. Yet, they were found to be less abundant in saliva^26^ and also less stable over time^33^. This is consistent with the observed baseline differences in the present study, with considerably higher spike- than RBD-specific sIgA (secretion rate: mean_spike-specific_ = 2.36, SD_spike-specific_ = 3.23; mean_RBD-specific_ = 1.05, SD_RBD-specific_ = 1.01). Also different from anti-spike, RBD-specific antibodies in saliva did not correlate well with RBD in serum^26^. The observed differences in the antibody response to the disease video might thus indicate some kind of compartmentalization of the mucosal immune response. In real life, the contagious respiratory droplets and aerosols of a sick person, that are emitted by sneezing, coughing, or even breathing, cannot be easily avoided in close social encounters. Thus, it may be adaptive to release the spike-specific sIgA as a proactive mechanism of immune exclusion, its release being already initiated in response to predictors of airborne contagion (here, the situations shown in the disease video). In contrast, the absence of an increase in RBD-specific sIgA in response to the visual disease predictors suggests, that the release of neutralizing antibodies may only be increased once the mucosae have come in contact with the viral antigen. This would then rather reflect a reactive immune response of the RBD-specific sIgA to the specific pathogen. The parallel decline of spike- and RBD-antibodies after the offset of the disease video, i.e., from Post-Video 1 to Post-Video 2, might then be explained by the discontinuation of the visual predictor (in case of anti-spike) and by the absence of a factual virus-mucosae contact (in case of anti-RBD, and supposedly also anti-spike), which would render an immune response unnecessary. However, future studies have to further address these speculations, especially those regarding the nature of the anti-RBD response and the proposed compartmentalization of the mucosal immune response.

From our present finding we cannot unequivocally infer that the mucosal immune response to the respiratory disease video will always follow the observed pattern. Even though, the shape of the spike-specific sIgA strongly resembled the one observed for total sIgA in our previous study^17^, and in the confirmatory analysis of the present study, our participants were nevertheless tested during the ongoing COVID-19 pandemic. Most tests of the current study took place during the first and second Omicron wave in Northern Germany. Although all participants were vaccinated, the new Omicron variant and its various subvariants created a context of heightened contagion risk for COVID-19, as seen by the large number of breakthrough infections among vaccinated individuals in 2022^34^. For other viral respiratory pathogens like influenza, a high risk context (e.g., the flu season) has previously been shown to be linked to a surge in total sIgA to visual disease predictors, while a low risk context was not ^13^. It remains to be ascertained in the future, how people would respond to our disease video once SARS-CoV-2 has become endemic and COVID-19 morbidity and mortality is significantly reduced.

Apart from the increase in spike-specific sIgA on the group level, there was also considerable variance in the extent of the ΔsIgA_spike_. Such interindividual differences in the proactive immune response have previously been explained by a compensatory relationship between physiological immune and behavioral avoidance responses. The associated feeling of disgust may thereby facilitate avoidance of disease cues, which in turn reduces the need to prepare the immune system for potential pathogen contact^17,35,36^. In line with these prior findings, we found an inverse correlation of ΔsIgA_spike_ with the *Composite Respiratory Score* from the *Interoceptive-Feelings Questionnaire*^20^. Interoception is a wide construct that not only includes the awareness/feeling of bodily sensations, but also the interpretation of such information and the consequential behavior^37^. Thus negative, oral and contamination-related interoception such as an itch in the throat, the urge to cover your mouth or the feeling of flu-like symptoms during the video can be seen as proactive interoceptive responses that may trigger avoidance of their generators. We did not find a significant correlation between ΔsIgA_spike_ and the *Composite Gut Score*, and the respective score was lower than the *Composite Respiratory Score* (see SI Results 2.5). This indicates that acute bodily sensations may be specific for the category of disease cues and the associated pathway of contagion. COVID-19 is mainly a respiratory disease^2^, and airborne transmission is the dominant route of contagion^38^, which is why the present disease video, that focused on respiratory symptoms, may have specifically triggered sensations in the respiratory pathway. In a similar vein, we observed an inverse correlation of ΔsIgA_spike_ and self-reported state disgust experienced during the disease video. While this also fits with the hypothesis of a compensatory relationship between behavioral and physiological responses to enhanced contagion risk^39^, this relationship has not been found with total sIgA^15,17,40^. We can only speculate that either the spike-specific sIgA surge is uncoupled from total sIgA in saliva, which is rather unlikely since our confirmatory analysis showed a correlation between the two, or that the current disease video induced a sufficient variation in both the state disgust rating and the physiological immune response of the 45 participants, rendering this correlation more likely. However, since all correlations were rather small (< 0.3), a replication is needed. What is nevertheless noteworthy is the complete absence of an association between ΔsIgA_spike_ and the trait measures of disgust and disease vulnerability. Like in our previous study^17^, these trait measures may not be indicative of the capacity of the mucosal immune system to proactively release antibodies in response to predictors of contagion.

On an intraindividual level we should note that the baselines of spike-specific and RBD-specific sIgA showed a slight variation between disease and control video, although not significant. As a highly variable parameter that responds to even small changes in the mouth (e.g., chewing^28^, food or drink^41^), sIgA baseline differences even within the same person (when tested on different days) were to be expected. Different from caged test animals, daily stressors, differences in food ingestion etc. could not be controlled in our human volunteers. Although we tested only nonsmokers, instructed our participants to refrain from eating 2 h before the test and to refrain from taking medication or food additives for at least 48 h before the test, we had no chance to control everything in their daily life.

In addition to that, we also explored the within-subject design for possible order effects and observed a significant three-way interaction between the factors *Video, Sample* and *Video Order* in the spike-specific sIgA, which also became evident in the analysis of total sIgA (see SI Results 2.4.3). The increase of spike-specific sIgA during the disease video thereby only differed significantly from the change during the control video, if participants experienced the disease video first. We can only speculate that this may have been caused by an interpretational bias. Interpretational biases in (visual) cognition have already been found to alter emotional reactions and may possibly also affect the associated physiological responses^42,43^. The present study was explicitly advertised as a project that assessed immunological responses to SARS-CoV-2. This advertisement might have led to certain expectancies that should have particularly affected the naïve test day, when everything was new and participants expected to contribute in a research project on SARS-CoV-2. As a result, watching the disease video on the naïve test day might have induced a potent effect on sIgA release, while even the control video might have been perceived as more salient on the first day, also given common knowledge that even asymptomatic persons can transmit the virus^44^. In an explorative comparison we found that participants perceived the control video as more disgusting when they saw the control video first (see SI Results 2.4.4.). Then, on the second day, the reduced relative rise during the disease video may also be explained an expectation effect. After having watched the control video on the first day, participants most likely expected to receive a more disease-associated stimulation on day 2, which would fit with the observation of the already higher spike-specific sIgA baseline concentration on the second day in the group of participants that watched the control video first (see SI Fig. S3b). However, since this order effect was analyzed post-hoc, we can only speculate in this regard. Future studies will be necessary to assess the influence of interpretational biases and expectancy effects on proactive immunological responses, which might be caused by prior experience, task order or conditioning effects. Finally, it is important to note that our pre-registered study design was counterbalanced for task order and we also found a significant increase in both spike-specific and total sIgA after the disease video in the total group, regardless of video order.

This is the first study that demonstrated the plasticity of salivary antibody levels against SARS-CoV-2 in response to a visual simulation of heightened airborne contagion potential. It shows that spike-specific sIgA can be released on demand, in response to unequivocal disease cues and at one of the crucial viral entry points, the oral mucosae. Nevertheless, several important questions still remain unanswered. First, the virus neutralizing capacity of the released spike-specific sIgA was not tested, and therefore the actual immunological advantage of this proactive response remains to be proven. Second, the meaning of the decline in RBD-specific sIgA could only be indirectly attributed to the absence of a factual viral exposure, and the interpretation of this finding thus rather represents a hypothesis than an inference. Again, further evidence is needed to probe the theory that neutralizing RBD-antibodies require mucosal contact with the virus to be released. Third, as already indicated above, this study was conducted during the COVID-19 pandemic in a phase of heightened contagion risk, i.e., Omicron waves. In addition, the participants had quite recently received a vaccination, which was also reflected by the relatively high average blood IgG-titer that may be associated with a potent mucosal antibody reservoir. For these reasons, our results might be quite specific for the pandemic situation and a population with sufficient immunity. It thus needs to be ascertained in the future, whether these results of our intervention can be replicated in people with dwindling antibody levels and outside of the pandemic context. Finally, our study does not answer the question, whether less obvious markers of respiratory diseases (e.g., changes in skin coloration, increased sweating) that may be carried by otherwise asymptomatic people, and which might be unconsciously perceived^45^, also have the potential to activate this route of the mucosal immune defense. In that context, the associated neural pathway would also be of increased interest.

## Materials and methods

### Participants

In a within-subject design we confronted the participants with two different videos (disease and control) on two different test days. We recruited 46 participants (24 m/22 f) on the university campus, through online advertisements, and via social media. We only invited healthy individuals to participate, who were between 18 and 35 years old, and who had been vaccinated at least twice with one of the mRNA-vaccines against SARS-CoV-2. Female participants were only included, if they used hormonal contraception containing ethinylestradiol (to ensure a homogeneity of steroid hormones within the female participants). Data collection took place from February to April 2022. Participants received a financial reward of 35 Euros. We obtained informed consent from all participants and the procedure was approved by the local ethics committee *“Ethikkommission der Ärztekammer Hamburg”* (PV3938) and conformed with the Declaration of Helsinki.

### Stimuli

**Figure 4:**
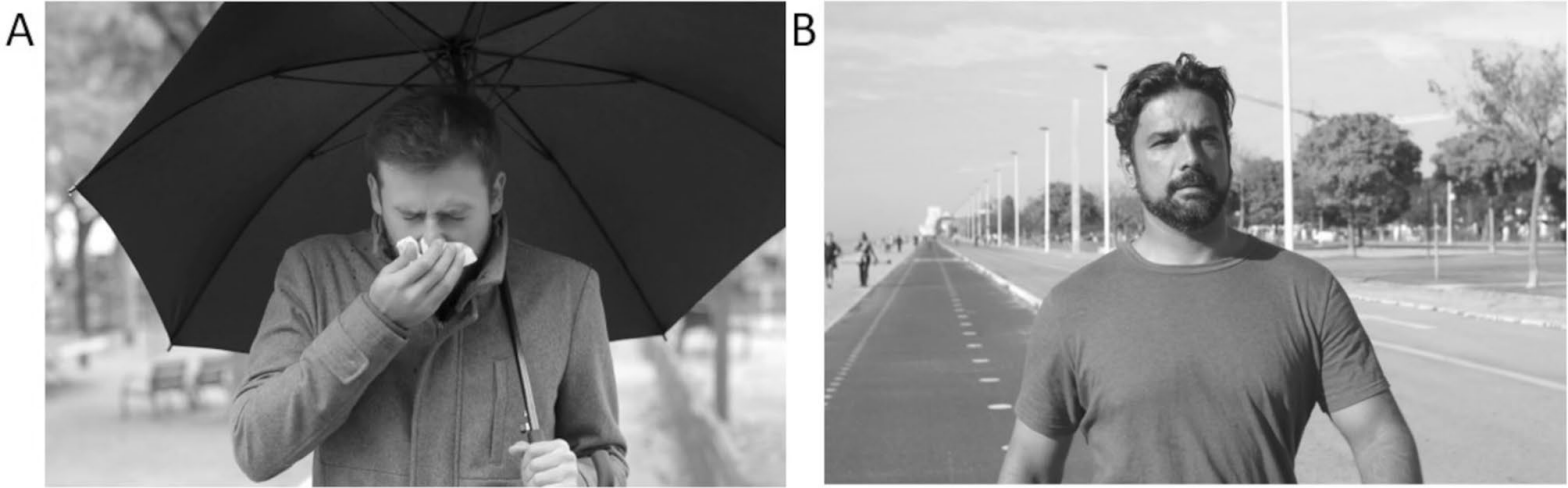
Examples from the two stimulus sets used in the videos. (**A**) Exemplary screenshot from the disease video (www.istockphoto.com; by Antonio Guillem); (**B**) Exemplary screenshot from the control video (www.pexels.com; by Kampus Production).

During the two test sessions, participants were primed with either a disease or a control video. The order of the videos was counterbalanced. The disease video was a 5 min video displaying short clips of people with symptoms of respiratory diseases, e.g., sneezing and coughing, as well as blowing their nose and lying sick in bed (Fig. 4a). The control video was matched to the disease video and showed healthy people in similar environments (Fig. 4b). User licenses for videos were obtained from the respective online platforms (iStock, pexels, etc.). For detailed information see SI Tables S2 & S3.

### Procedure

Prior to invitation for test sessions, all participants completed an online survey on demographic data and medical history. This survey also included the revised *Disgust Scale* (DS-R)^19^ as well as the *perceived Vulnerability to Disease Questionnaire* (pVtD)^18^.

The two test sessions were conducted at the Institute for Animal Cell and Systems Biology, Universität Hamburg in the afternoon (between 12 and 5 pm, and at least 24 h apart [x̄ = 5.56 days; σ = 4.39 days]). In the beginning of the first test session, participants were informed about the general purpose of the study, the opportunity to abort data collection at any time, as well as aspects concerning anonymity and safety. Upon arrival, participants also gave an initial practice saliva sample that was discarded afterwards. Subsequently, they watched a 5 min relaxation video showing waterfalls and nature scenery, while listening to relaxing music. The relaxation video was intended to reduce anticipatory stress and anxiety in the unknown test environment. This was followed by the Baseline saliva sample and participants providing additional demographic data on aspects such as age, sex, and current state of health. Here, they also reported, whether they had been exposed to any stressors, such as smoking, sports, alcohol within the last 48 h, as well as any current and previous diseases, before moving on to the *Mood Scale*^46^. The *Mood Scale* was included to control for potential mood differences between test days. It was followed by one of the two videos, to which the participants were randomly assigned on the first test day (the other video was shown on the second test appointment). The second saliva sample was taken immediately after the end of the video (Post-Video 1). After filling out further questionnaires related to attention, emotion^21^ and somatic feelings during the video stimulation, participants were finally asked to give the third and last saliva sample (Post-Video 2).

At the end of the disease video session, we finally measured participants’ IgG-Titer in the blood (BAU/mL) utilizing the VitaLab LS-1100 diagnostic device with the dry fluorescence Immunoassay Test Kit.

### Saliva samples

During each test session three saliva samples were collected at Baseline, Post-Video 1 and Post-Video 2 (Fig. 1a). Participants filled the three microcentrifuge tubes (2 mL) by passive drooling. The experimenter stopped the time it took to fill up a tube. Afterwards the samples were weighed and frozen at − 80 °C. After being frozen for at least 24 h the samples were thawed and deactivated (centrifuged, mixed with tri-n-butyl phosphate and Triton-X100), as per protocol in Becker et al.^8^. Salivary IgA titers were analyzed using MULTICOV-AB, a multiplex SARS-CoV-2 immunoassay^8,47^ to determine SARS-CoV-2 antigen-specific antibody titers and an IgA ELISA (LDN Immunoassays #SA E-6800R) to determine total salivary IgA. Both protocols were performed either according to the manufacturer’s protocol (IgA ELISA) or as previously described (MULTICOV-AB), whereby each saliva sample was assayed twice and the mean of the two measurements was used for analysis. All saliva analysis were performed blinded, although all samples from a single individual were included on the same plate. The values of the Sars-CoV-2 specific sIgA were normalized to nucleocapsid antibodies. Normalization was performed to standardize and remove as many environmental effects as possible. This is often necessary with saliva due to the inherent material itself. Saliva is not an ideal matrix due to the number of individual differences present (e.g. viscosity, bacterial/yeast contamination), all of which affect the ability to generate accurate measurements from it. While normalization would usually involve the use of reference samples, unfortunately saliva reference samples were unavailable due to the type of material itself, making this type of normalization impossible. Similarly, normalizing to reference serum samples would not have been ideal, as our normalization values would have then been resulting from a completely different sample matrix. We therefore chose to normalize between analytes in a sample as is done for other molecular biology techniques such as RT-PCR. This enabled us not only to have a direct evaluation of the change in antibodies generated/detected (e.g. increase in spike production), but also to normalize our samples regardless of their individual differences. By using Nucleocapsid antibodies as an effective quality control from sample to sample, we could assess antigen-specific changes in antibody levels within each sample.

### Data analysis

After data collection, but before data analysis, we preregistered the planned analysis (https://osf.io/br3xm/). For data analysis we calculated the sIgA secretion rate, which is determined by multiplying the absolute sIgA Measure (normalized ($\frac{Spike or RBD}{Nucleocapsid}$) mean fluorescence intensity (MFI)) with the flow rate ($\frac{mL}{min}$) (i.e., secretion rate = $\mathrm{normalized MFI}* \frac{mL}{min}$). All data were tested for deviation from a normal distribution using the Kolmogorov–Smirnov test with a statistical threshold of *p* < 0.05. Since all KS-tests were significant, we used non-parametric post-hoc tests. All data analysis was conducted with IBM SPSS (Version 29.0.0.), figures were generated using R Studio (Version 4.2.3).

We assessed whether the increase in sIgA secretion rate (spike- and RBD-specific) was affected by the category of the videos. For this, we planned to utilize a 2 × 3 general linear model for repeated measures (GLM) with *Video* (disease and control video) and *Saliva Sample* (Baseline, Post-Video 1, and Post-Video 2) as within-subject factors. However, during the analysis process, we decided to use a Generalized Linear Mixed Model (GLMM), which allows adding random effects of intercept as well as of slopes. As the distribution of sIgA data has a left skew and no negative values we decided to use a gamma distribution with log link, with sIgA secretion rate as Target, *Video* & *Sample* as Fixed Effects and Interactions and a random intercept of *Subject.* We further utilized robust covariances to accommodate for possible violations of model assumptions (the SPSS syntax file is uploaded under  https://osf.io/br3xm/). The results of the originally planned GLM can further be found in the Supplement (see SI Results 2.6). As post-hoc tests, we conducted Wilcoxon-signed-rank tests. In addition, we employed Spearman-correlations to assess the association between the increase of sIgA (ΔsIgA = Post-Video1—Baseline) following the disease video and the questionnaire scores. Post-hoc test and correlations regarding our directed hypotheses were conducted one-sided.

### Supplementary Information

Supplementary Information.

## Data Availability

The data used for the analysis that support the findings of this study are available on OSF.io (https://osf.io/br3xm/).
