# Supplementary material for: SARS-CoV-2 specific sIgA in saliva increases after disease-related video stimulation
Source: Sci Rep. 2023 Dec 20;13:22631. doi: 10.1038/s41598-023-47798-y (PMC10733377; doi:10.1038/s41598-023-47798-y)
Supplement: Supplementary file 1 — Supplementary Information. [file 41598_2023_47798_MOESM1_ESM.pdf]

1. Supplementary Material & Methods

1.1 Participants

Table S1: Information about participants including Sex (f=female, m=male), Age (years), SARS-CoV-2 vaccination (Dose 1-3) and infection background of participants (reported cases confirmed by PCR)

ID	*Sex*	*Age*	*1st Dose*	*2nd Dose*	*3rd Dose*	*Previously infected*
1	m	24	Vaxzevria	BNT-162b2	BNT-162b2	No
2	f	21	BNT-162b2	BNT-162b2		Yes
3	m	30	mRNA-1273	mRNA-1273		Yes
4	f	25	BNT-162b2	BNT-162b2	BNT-162b2	No
5	m	25	BNT-162b2	BNT-162b2	mRNA-1273	No
6	m	21	BNT-162b2	BNT-162b2	BNT-162b2	No
7	m	22	BNT-162b2	BNT-162b2	BNT-162b2	No
8	m	29	Vaxzevria	BNT-162b2	BNT-162b2	Yes
9	f	26	BNT-162b2	BNT-162b2	BNT-162b2	No
10	m	25	BNT-162b2	BNT-162b2	BNT-162b2	No
11	m	20	BNT-162b2	BNT-162b2	BNT-162b2	No
12	m	26	BNT-162b2	BNT-162b2	BNT-162b2	No
13	f	24	BNT-162b2	BNT-162b2	BNT-162b2	No
14	f	20	BNT-162b2	BNT-162b2		Yes
15	f	28	BNT-162b2	BNT-162b2	BNT-162b2	No
16	m	26	mRNA-1273	mRNA-1273		Yes
17	f	23	mRNA-1273	mRNA-1273	mRNA-1273	Yes
18	m	24	BNT-162b2	BNT-162b2	BNT-162b2	Yes
19	m	32	mRNA-1273	mRNA-1273	BNT-162b2	No
20	f	18	BNT-162b2	BNT-162b2	BNT-162b2	No
21	f	24	mRNA-1273	mRNA-1273	BNT-162b2	No
22	f	24	BNT-162b2	BNT-162b2	BNT-162b2	No
23	m	24	BNT-162b2	BNT-162b2	BNT-162b2	No
24	m	27	BNT-162b2	BNT-162b2		Yes
25	m	19	BNT-162b2	BNT-162b2	BNT-162b2	No
26	f	20	BNT-162b2	BNT-162b2		No
27	m	30	BNT-162b2	BNT-162b2	mRNA-1273	No
28	f	33	BNT-162b2	BNT-162b2	mRNA-1273	No
29	m	29	BNT-162b2	BNT-162b2	BNT-162b2	No
30	m	20	BNT-162b2	BNT-162b2	BNT-162b2	No
31	f	20	BNT-162b2	BNT-162b2	BNT-162b2	No
32	f	30	BNT-162b2	BNT-162b2	mRNA-1273	No
33	f	28	BNT-162b2	BNT-162b2	BNT-162b2	Yes
34	f	23	BNT-162b2	BNT-162b2	BNT-162b2	No
35	m	35	Jcovden	mRNA-1273	mRNA-1273	No
36	m	28	BNT-162b2	BNT-162b2	BNT-162b2	No

Continuation of Table S1: Information about participants including Sex (f=female, m=male), Age (years), SARS-CoV-2 vaccination (Dose 1-3) and infection background of participants (only cases confirmed by PCR)

Subject	*Sex*	*Age*	*1st Dose*	*2nd Dose*	*3rd Dose*	*Previously infected*
37	m	28	mRNA-1273	mRNA-1273	BNT-162b2	No
38	f	24	BNT-162b2	BNT-162b2	mRNA-1273	No
39	f	21	BNT-162b2	BNT-162b2	BNT-162b2	No
40	f	31	BNT-162b2	BNT-162b2	BNT-162b2	No
41	f	20	BNT-162b2	BNT-162b2	BNT-162b2	No
42	f	26	BNT-162b2	BNT-162b2	BNT-162b2	No
43	m	33	BNT-162b2	BNT-162b2	BNT-162b2	No
44	f	23	Jcovden	BNT-162b2	BNT-162b2	No

1.2 Video material

Table S2: Content of the disease video. Sequence (Seq.), length and description of the video content shown.

Seq.	*Length (s)*	*Description*
1	3	Man, facing camera, sneezing
2	8	Woman, side profile, slow motion, sneezing, aerosols visible
3	12	Woman, sick in bed, coughing into tissue
4	10	Woman, facing camera, slow motion, sneezing, aerosols and snot visible
5	8	Woman, facing camera, sneezing 3 times, teary eyes
6	22	Man, facing camera, walking with an umbrella, sneezing into tissue 4 times, red nose
7	4	Man, facing camera, slow motion, sneezing, aerosols visible, blurry
8	8	Man, facing camera, sneezing 3 times, teary eyes
9	14	Woman, lower half of face visible, sitting outside, cleaning nose with tissue
10	13	Man, facing camera, sneezing 4 times, drool visible, teary eyes
11	7	Woman, side profile, sneezing into tissue and cleaning nose
12	4	Man, facing camera, sneezing, tears and a lot of drool visible, teary eyes
13	7	Woman, facing camera, sneezing 3 times, teary eyes
14	3	Woman, facing camera, sneezing, teary eyes
15	12	Man, sitting outside, side profile, lower half of face visible, sneezing and coughing 2 times

Continuation of Table S2: Content of the disease video. Sequence (Seq.), length and description of the video content shown.

Seq.	*Length (s)*	*Description*
16	8	Man, side profile, slow motion, sneezing, a lot of drool and aerosols visible
17	6	Woman, facing camera, sneezing, drool visible, teary eyes
18	8	Man, facing camera, sneezing 2 times, aerosols and tears visible, teary eyes
19	6	Man, side profile, slow motion, outside, Sneezing, aerosols visible
20	4	Man, facing camera, slow motion, outside, sneezing, aerosols visible
21	3	Man, facing camera, slow motion, outside, sneezing, aerosols and snot visible
22	10	Woman, facing camera, sitting on bed, sneezing 6 times
23	7	Man, facing camera, slow motion, coughing
24	11	Man, facing camera, slow motion, sneezing, snot visible
25	4	Woman, facing camera, sneezing 2 times, teary eyes
26	13	Man, facing camera, sitting on couch, visibly sick, sneezing into tissue, drinking tea
27	11	A sequence of short clips showing 3 women and 6 man, facing camera, sneezing
28	6	Woman, side profile, slow motion, sneezing into tissue
29	3	Man, side profile, sneezing 2 times, aerosols visible
30	23	Man, side profile, slow motion, sneezing, a lot of aerosols visible
31	4	Man, facing camera, sneezing, tears and a lot of drool visible, teary eyes
32	7	Woman, facing camera, sneezing 2 times, aerosols visible
33	16	Man, facing camera, slow motion, sneezing
34	13	Woman, side profile, sick in bed, sneezing into tissue 2 times
total	**299**	

Table S3: Content of the control video. Sequence (Seq.), length and description of the video content shown.

Seq.	*Length*	*Description*
1	13	Woman, facing camera, looking at camera
2	10	Man, sitting on couch, talking on his phone
3	10	Woman, lying in bed
4	14	Man, facing camera, looking at the camera
5	6	Woman, facing camera, smiling at the camera
6	8	Man, top half of body visible, walking outside
7	18	Woman, lying in bed, stretching arms
8	8	Man, facing camera, looking around
9	11	Woman, side view, entire body visible, typing on the computer, smiling
10	9	Man, top half of body visible, typing on his phone, scratching his nose
11	11	Woman, facing camera, looking and smiling at camera
12	12	Man, facing camera, looking around, scratching his cheek
13	20	Woman, side view, entire body visible, outside, leaning against a railing, drinking out of a cup
14	14	Man, side view, entire body visible, sitting on a bed, typing on computer
15	4	Woman, facing camera, looking at the camera
16	7	Man, facing camera, looking and smiling at camera
17	16	Woman, side view, upper half of body visible, sitting on a bed, typing on computer
18	16	Man, facing camera, lying in bed with closed eyes, moving around
19	21	Woman, side view, entire body visible, sitting on an armchair, reading a book
20	10	Man, front view, entire body visible, sitting on a bed, typing on computer
21	5	Woman, facing camera, looking at the camera
22	6	Man, birds view, upside down, lying on the floor, listening and moving to music
23	21	Woman, front view, upper half of body visible, leaning against kitchen counter, looking at phone, drinking out of a cup
24	13	Man, front view, upper half of body visible, walking through a glass building
25	10	Woman, side view, entire body visible, lying on bed, reading a book
total	**293**	

1.3 Description of Questionnaires

Table S4: Interoceptive Questionnaire that follows the Post-Video 1 sample (modified and adapted questionnaire of Kupfer et al. (2021); two subscales were formed by averaging corresponding questions)

**Composite score**	***Please rate the following statements:*** **(options: *1-not at all, 2, 3, 4, 5, 6, 7-very strongly*)**
**Gut**	*I felt nauseous during the video.*
**Gut**	*I felt like I could vomit during the video*.
**Gut**	*I felt a physical sensation in my stomach, during the video.*
**Respiratory**	*I felt a physical sensation in my throat.*
**Respiratory**	*I felt an increased saliva secretion during the video.*
**Respiratory**	*I felt an urge to cover my mouth and nose with my hand during the video.*
**Respiratory**	*I had a feeling of contamination during the video.*
**Respiratory**	*I felt unclean during the video.*
**Respiratory**	*I felt the urge to wash my hands during the video*.
**Respiratory**	*I felt slightly sore during the video.*
**Respiratory**	*I felt flu-like symptoms during the video.*

2. Supplementary Results

2.1 Evaluation of video content

The disease and control video were rated with regard to (a) their disgust potential (*"How strong was your feeling of disgust, antipathy and revulsion while watching the video?"*; Likert scale from 0="*not at all*" to 8="*completely*") and (b) the associated contagion risk ("*During the video I had the feeling that I could get infected*"; Likert scale from 0="*completely disagree*" to 7="*strongly agree*"). Utilizing a Wilcoxon signed-rank test, we found that the disease video was on average rated as significantly more disgusting ($z=-5.80$, $p<.001$, $\eta^2=.75$) and more contagious ($z=-4.56$, $p<.001$, $\eta^2=.46$) than the control video.

In a more fine-grained rating of disgust we showed participants single screenshots of the situations shown in the video. Although this rating replicated the significant difference between the videos ($z=5.84$, $p<.001$, $\eta^2=.76$), which we already documented for the broader disgust rating that referred to the whole video, there was also some unexpected interindividual variance in the screenshot disgust rating of the control video (see Figure S1), which led us to perform the exploratory analysis in 2.4.1.1

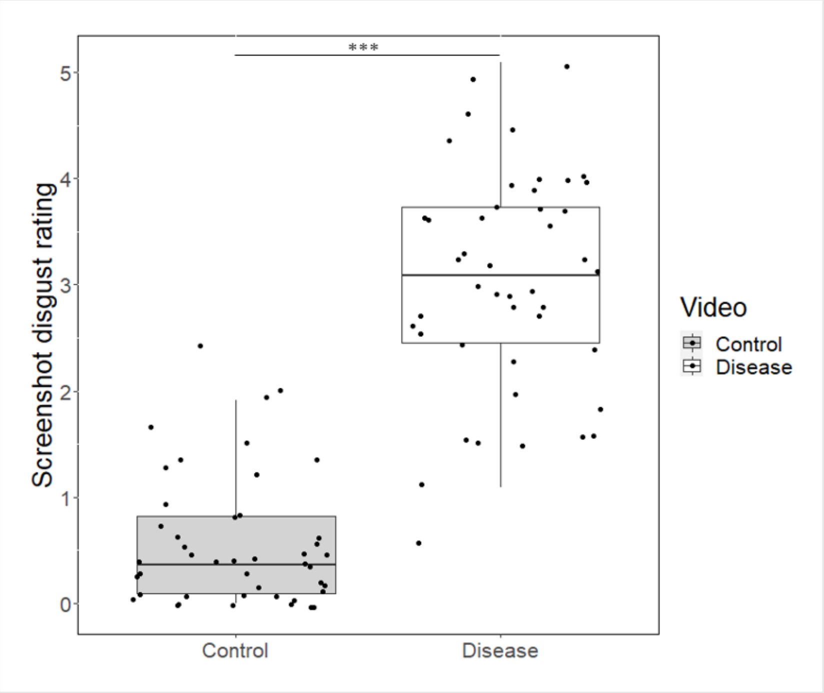

Figure S1: Screenshot disgust rating of control (grey) and disease (white) video. Box represents the median and the 25th and 75th percentiles, whiskers the smallest and the largest value or no further than 1.5*IQR (inter-quartile range). Significant differences are marked with asterisks (***p<.001).

2.2 GLMM Model Coefficients

Table S5: Fixed Coefficient of spike-specific sIgA secretion model with the Fixed Factors *Video* and *Sample* and their Interaction. With Coefficient (β), Standard Error of Coefficient (SE_β), t-value, p-value and lower as well as upper Confidence Intervals (CI).

Model Term	β	SE_β	t	p	95% CI	
					Lower	Upper
Intercept	.05	.18	.29	.773	-.29	.40
Video=Disease	.42	.16	2.58	.010	.10	.74
Video=Control	0[a]					
Sample=Post-Video 2 (PV2)	-.12	.11	-1.06	.291	-.35	.10
Sample=Post-Video 1 (PV1)	-.13	.11	-1.22	.224	-.34	.08
Sample=Baseline	0[a]					
[Video=Disease]*[Sample=PV2]	-.05	.18	-.25	.799	-.41	.31
[Video=Disease]*[Sample=PV1]	.34	.15	2.29	.023	.05	.64
[Video=Disease]*[Sample=Baseline]	0[a]					
[Video=Control]*[Sample=PV2]	0[a]					
[Video=Control]*[Sample=PV1]	0[a]					
[Video=Control]*[Sample=Baseline]	0[a]					

[a]This coefficient is set to zero because it is redundant.

Table S6: Fixed Coefficient of RBD-specific sIgA secretion model with the Fixed Factors *Video* and *Sample* and their Interaction. With Coefficient (β), Standard Error of Coefficient ($SE_β$), t-value, p-value and lower as well as upper Confidence Intervals (CI).

Model Term	β	$SE_β$	t	p	95% CI	
					Lower	Upper
Intercept	.87	.09	9.33	<.001	.69	1.05
Video=Disease	.16	.09	1.85	.066	-.01	.33
Video=Control	0[a]					
Sample=Post-Video 2 (PV2)	.35	.15	2.41	.017	.06	.64
Sample=Post-Video 1 (PV1)	.20	.09	2.29	.023	.03	.38
Sample=Baseline	0[a]					
[Video=Disease]*[Sample=PV2]	-.50	.15	-3.33	.001	-.79	-.20
[Video=Disease]*[Sample=PV1]	-.34	.11	-3.16	.002	-.56	-.13
[Video=Disease]*[Sample=Baseline]	0[a]					
[Video=Control]*[Sample=PV2]	0[a]					
[Video=Control]*[Sample=PV1]	0[a]					
[Video=Control]*[Sample=Baseline]	0[a]					

[a]This coefficient is set to zero because it is redundant.

2.3 Confirmatory analysis of total sIgA

In order to confirm that the present disease video had a comparable effect on total sIgA as the disease videos used in our previous study (Keller et al., 2022), we additionally analysed the saliva samples for the content of total sIgA (Please note, in this analysis we had to exclude an additional participant as he was an outlier with regard to the total secretion rate of all 6 saliva samples.). The confirmatory analysis was performed, because the videos from our previous study showed some differences from the presently employed stimulation. The first disease video of our previous only study showed sneezing or coughing people, who often visibly spread aerosols or droplets and never covered their nose or mouth, while the second disease video of that prior study used concealed contagion stimuli such as people lying sick in bed or sneezing into a tissue. Further, both previous disease videos were mute. The presently used disease video showed a mixture of content displaying openly and concealed contagious persons and also contained the sneeze and cough audios for a more realistic stimulation. The control video from our prior study also differed in some important aspects. It primarily showed landscape or street impressions, which seldomly included people, and if so, people were shown only from a

distance and the video never focused on a certain person, whereas the present control video showed healthy people in everyday situations.

In the GLMM with total sIgA we found a significant main effect if *Sample* ($F_{(2,258)}$=13.39, p<.001) as well a significant interaction between *Sample* and *Video* ($F_{(2,258)}$=4.35, p=.014), but no significant main effect of *Video* ($F_{(1,258)}$=1.61, p=.205, please find fixed coefficients in Table S7). In the post-hoc tests, we found a significant difference between the Baseline and the Post-Video 1 sample after watching the disease prime (z=-4.49, p<.001, η^2=.46), as well as after watching the control prime (z=-2.22, p=.027, η^2=.11). However, the increase ($\Delta sIgA_{total}$) after the disease video was significantly higher than the one after the control video (z=-1.75, p=.040, η^2=.07, see Figure S2a). Furthermore, the samples Post-Video 1 and 2 differed significantly after disease video (z=-3.28, p=.001, η^2=.25) but not after the control video (z=-.44, p=.657) (see also Figure S2b).

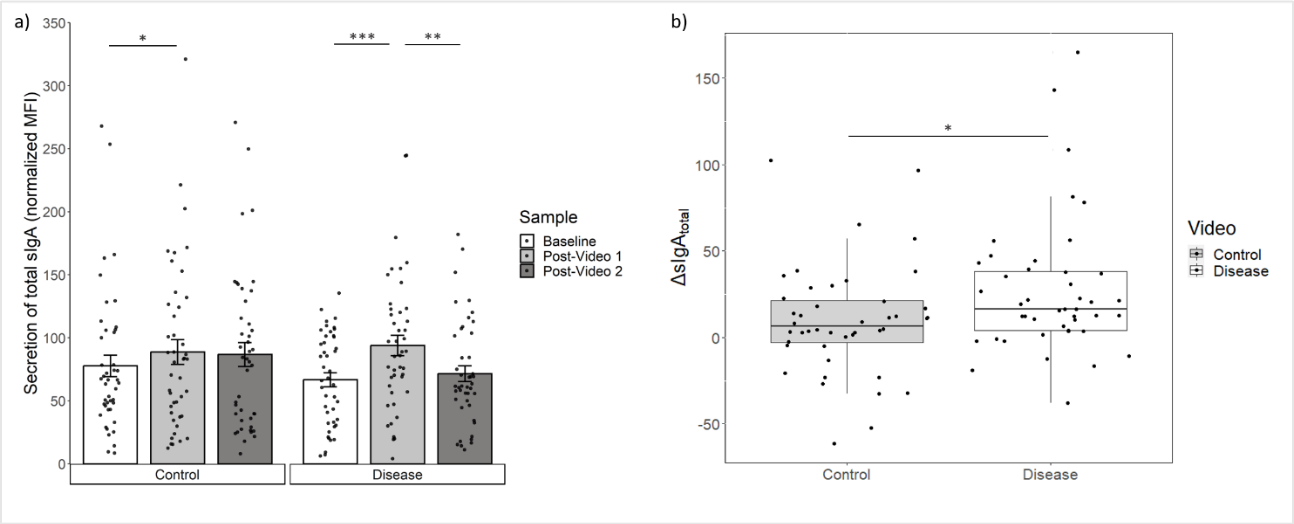

Figure S2: a) Change in total sIgA for disease and control video intervention. Mean, standard errors and individual data points of the secretion rate at Baseline, directly after the video (Post-Video 1), and several minutes after the video (Post-Video 2). Significant changes are marked with asterisks (*p<.05; ***p<.001). b) $\Delta sIgA_{total}$ of the control (grey) and disease (white) video. Box represents the median and the 25th and 75th percentiles, whiskers the smallest and the largest value or no further than 1.5*IQR (inter-quartile range). Significant differences are marked with asterisks (*p<.05).

Table S7: Fixed Coefficient of total sIgA secretion model with the Fixed Factors *Video* and *Sample* and their Interaction. With Coefficient (β), Standard Error of Coefficient ($SE_β$), t-value, p-value and lower as well as upper Confidence Intervals (CI).

Model Term	β	$SE_β$	t	p	95% CI	
					Lower	Upper
Intercept	4.18	.10	40.20	<.001	3.98	4.39
Video=Disease	-.10	.11	-.96	.337	-.31	.11
Video=Control	0[a]					
Sample=Post-Video 2 (PV2)	.07	.08	.85	.396	-.09	.22
Sample=Post-Video 1 (PV1)	.08	.06	1.16	.247	-.05	.20
Sample=Baseline	0[a]					
[Video=Disease]*[Sample=PV2]	-.01	.12	-.11	.931	-.24	.22
[Video=Disease]*[Sample=PV1]	.26	.10	2.73	.007	.07	.46
[Video=Disease]*[Sample=Baseline]	0[a]					
[Video=Control]*[Sample=PV2]	0[a]					
[Video=Control]*[Sample=PV1]	0[a]					
[Video=Control]*[Sample=Baseline]	0[a]					

[a]This coefficient is set to zero because it is redundant.

2.4 Explorative analysis of Video Order

2.4.1 Spike-specific sIgA

As an explorative analysis, we ran a second GLMM on spike-specific sIgA with the covariate *Video Order*. This was done, because the first test day, and the type of video one had watched, may have had an influence on spike-specific sIgA secretion on the second test day. We found that the two-way interaction between *Video* and *Sample* was still significant ($F_{(2,258)}$=6.60, p=.002), while the main effects of *Video* ($F_{(1,258)}$=.70, p=.403) and *Sample* ($F_{(2,258)}$=.90, p=.409) were not significant anymore. The additional factor *Video Order* ($F_{(1,258)}$=.23, p=.636) and its two two-way interactions with *Video* Video Order* ($F_{(1,258)}$=.33, p=.565) and *Sample* Video Order* ($F_{(2,258)}$=.701, p=.497) were also not significant. However, the three-way interaction of *Video*Sample* Video Order* ($F_{(2,258)}$=7.33, p<.001) was significant (please find fixed coefficients in Table S9).

When data was split according to Video Order, post-hoc tests on ΔsIgA showed that the increase between Baseline and Post-Video 1 was only significantly higher after the disease video compared to the control video, when participants watched the disease video first (z=-2.71,

p=.007, η^2=.16), but not when they watched the control video on day one (z=1.43, p=.153). Within the participants, who watched the disease video first, we found similar results as in the analysis across the whole group: Accordingly, spike-specific sIgA at Post-Video 1 was significantly higher when having watched the disease video (z=-2.58, p=.010, η^2=.30). There was also a significant difference between Baseline and Post-Video 1 after the disease video (z=-3.00, p=.003, η^2=.41). In participants, who watched the Control video first, we found a significant difference between the Baselines (z=-2.80, p=.005, η^2=.34) and the Post-Video 1 sample (z=-2.01, p=.045, η^2=.18) between the two days. As well as a significant difference between and Post-Video 2 Sample and Baseline (z=-2.46, p=.014, η^2=.26) as well as Post-Video 1 Sample (z=-1.98, p=.048, η^2=.17) on the second day, when participants watched the disease video (also see Tabel S8 and Figure S3).

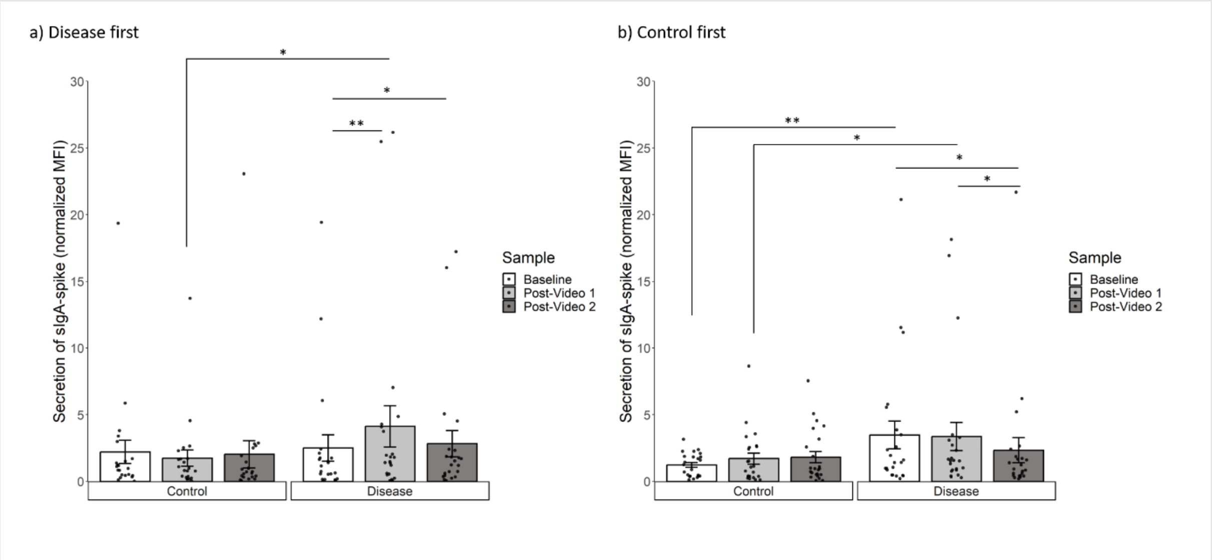

Figure S3: Bar plots with mean, standard errors and individual data points of the spike-specific sIgA secretion rate at Baseline, directly after the video (Post-Video 1), and several minutes after the video (Post-Video 2). For (a) participants (n=22) who watched the disease video first and (b) participants (n=23) who watched the control video first. Significant changes are marked with asterisks (*p<.05; **p<.01), based on Wilcoxon signed rank test.

Table S8: Post-hoc results of Wilcoxon signed rank test wit z values and p-values in parenthesis, comparing spike-specific sIgA at Baseline, Post-Video 1 (PV1) and Post-Video 2 (PV2).

**Video Order**	**Disease**			**Control**			**Comparison of Videos**		
	Baseline vs. PV1	Baseline vs. PV2	PV1 vs. PV2	Baseline vs. PV1	Baseline vs. PV2	PV1 vs. PV2	Baselines	Post-Video 1	Post-Video 2
Disease first	-3.00 (.003)	-.70 (.485)	-1.74 (.082)	-.63 (.527)	-1.22 (.223)	-.503 (.615)	-.70 (.485)	-2.58 (.010)	-1.64 (.101)
Control first	-.55 (.584)	-2.46 (.014)	-1.98 (.048)	-1.40 (.162)	-1.46 (.144)	-.58 (.563)	-2.80 (.005)	-2.01 (.045)	-.12 (.903)

Table S9: Fixed Coefficient of spike-specific sIgA secretion model with the Fixed Factors *Video*, *Sample* and *Video Order* and their Interaction. With Coefficient (β), Standard Error of Coefficient ($SE_β$), t-value, p-value and lower as well as upper Confidence Intervals (CI).

Model Term	β	$SE_β$	t	p	95% CI	
					Lower	Upper
Intercept	.27	.60	.45	.653	-.91	1.45
Video=Disease (D)	-.88	.54	-1.63	.104	-1.94	.18
Video=Control (C)	0[a]					
Sample=Post-Video 2 (PV2)	-.77	.34	-2.26	.025	-1.44	-.10
Sample=Post-Video 1 (PV1)	-.58	.34	-1.68	.094	-1.25	.10
Sample=Baseline	0[a]					
[Video=Disease]*[Sample=PV2]	1.79	.56	3.21	.001	.69	2.89
[Video=Disease]*[Sample=PV1]	1.76	.51	3.48	.001	.77	2.76
[Video=Disease]*[Sample=Baseline]	0[a]					
[Video=Control]*[Sample=PV2]	0[a]					
[Video=Control]*[Sample=PV1]	0[a]					
[Video=Control]*[Sample=Baseline]	0[a]					
Video_order	-.15	.34	-.44	.657	-.83	.53
Video_order*[Video=Disease]	.84	.31	2.74	.007	.24	1.44
Video_order*[Video=Control]	0[a]					
Video_order*[Sample=PV2]	.44	.21	2.05	.041	.02	
Video_order*[Sample=PV1]	.31	.20	1.51	.132	-.09	.70
Video_order*[Sample=Baseline]	0[a]					
Video_order*[Video=D]*[Sample=PV2]	-1.20	.33	-3.62	<.001	-1.86	-.55
Video_order*[Video=D]*[Sample=PV1]	-.92	.28	-3.30	.001	-1.47	-.37
Video_order*[Video=D]*[Sample=Baseline]	0[a]					
Video_order*[Video=C]*[Sample=PV2]	0[a]					
Video_order*[Video=C]*[Sample=PV1]	0[a]					
Video_order*[Video=C]*[Sample=Baseline]	0[a]					

[a]This coefficient is set to zero because it is redundant.

2.4.2 RBD-specific sIgA

When running the same explorative analysis with the covariate of *Video Order* on RBD-specific sIgA, We found that neither the two-way interaction between *Video* and *Sample* was still significant ($F_{(2,258)}$=.48, p=.620), nor the main effect of *Sample* ($F_{(2,258)}$=.90, p=.409) were significant. However, the main effect of *Video* ($F_{(1,258)}$=7.57, p=.001) was now significant. The additional main effect of *Video Order* ($F_{(1,258)}$=.24, p=.623) and its the twoway interaction of *Sample*Video Order* ($F_{(2,258)}$=.27, p=.764) was not significant. The interaction of *Video*Video Order* ($F_{(1,258)}$=13.80, p<.001) was however significant, while the three-way interaction of

*Video*Sample*Video Order* ($F_{(2,258)}$=1.04, p=.356) was not (find fixed coefficients in Table S10).

Table S10: Fixed Coefficient of RBD-specific sIgA secretion model with the Fixed Factors *Video*, *Sample* and *Video Order* and their Interaction. With Coefficient (β), Standard Error of Coefficient (SE_β), t-value, p-value and lower as well as upper Confidence Intervals (CI).

Model Term	β	SE_β	t	p	95% CI	
					Lower	Upper
Intercept	.54	.29	1.85	.066	-.04	1.41
Video=Disease (D)	.63	.31	2.02	.044	.02	1.24
Video=Control (C)	0[a]					
Sample=Post-Video 2 (PV2)	-.12	.52	-.22	.823	-1.13	.90
Sample=Post-Video 1 (PV1)	.12	.27	.46	.646	-.40	.65
Sample=Baseline	0[a]					
[Video=Disease]*[Sample=PV2]	.07	.48	.15	.884	-.88	1.02
[Video=Disease]*[Sample=PV1]	-.29	.37	-.77	.441	-1.03	.45
[Video=Disease]*[Sample=Baseline]	0[a]					
[Video=Control]*[Sample=PV2]	0[a]					
[Video=Control]*[Sample=PV1]	0[a]					
[Video=Control]*[Sample=Baseline]	0[a]					
Video_order	.22	.18	1.20	.233	-.14	.58
Video_order*[Video=Disease]	-.31	.17	-1.85	.065	-.64	.02
Video_order*[Video=Control]	0[a]					
Video_order*[Sample=PV2]	.31	.29	1.06	.290	-.27	.89
Video_order*[Sample=PV1]	.05	.18	.30	.763	-.29	.40
Video_order*[Sample=Baseline]	0[a]					
Video_order*[Video=D]*[Sample=PV2]	-.37	.29	-1.28	.202	-.95	.20
Video_order*[Video=D]*[Sample=PV1]	-.04	.22	-.16	.870	-.47	.39
Video_order*[Video=D]*[Sample=Baseline]	0[a]					
Video_order*[Video=C]*[Sample=PV2]	0[a]					
Video_order*[Video=C]*[Sample=PV1]	0[a]					
Video_order*[Video=C]*[Sample=Baseline]	0[a]					

[a]This coefficient is set to zero because it is redundant.

2.4.3 Total sIgA

Lastly we ran this explorative analysis with the covariate of *Video Order* on total sIgA, we found that the two-way interaction between *Video* and *Sample* ($F_{(2,252)}$=5.04, p=.005) and the main effect of *Sample* ($F_{(2,252)}$=5.41, p=.007) were still significant. The main effect *Video* ($F_{(1,252)}$=.126, p=.722) was still not significant. The additional factor *Video Order* ($F_{(1,252)}$<.01,

p=.961) and the two-way interactions of *Video*Video Order* ($F_{(1,252)}$<.01, p=.970) and *Sample*Video Order* ($F_{(2,252)}$=1.712, p=.183) were also not significant. However, the three-way interaction of *Video*Sample*Video Order* ($F_{(2,252)}$=5.00, p=.007) was significant (find fixed coefficients in Table S11). When data was split by *Video Order*, the post-hoc test on ΔsIgA showed that the increase between Baseline and Post-Video 1 was only significantly higher after the disease video compared to the control video, when participants watched the disease video first (z=-2.19, p=.028, η^2=.12), but not when they saw the control video first (z=-.21, p=.833).

Table S11: Fixed Coefficient of total sIgA secretion model with the fixed factors *Video*, *Sample* and *Video Order* and their Interaction. With Coefficient (β), Standard Error of Coefficient (SE_β), t-value, p-value and lower as well as upper Confidence Intervals (CI).

Model Term	β	SE_β	t	p	95% CI	
					Lower	Upper
Intercept	4.39	.31	13.95	<.001	3.77	5.01
Video=Disease (D)	-.92	.34	-2.72	.007	-1.58	-.25
Video=Control (C)	0[a]					
Sample=Post-Video 2 (PV2)	-.30	.25	-1.22	.223	-.78	.18
Sample=Post-Video 1 (PV1)	-.10	.21	-.48	.631	-.51	.31
Sample=Baseline	0[a]					
[Video=Disease]*[Sample=PV2]	1.06	.35	3.04	.003	.37	1.74
[Video=Disease]*[Sample=PV1]	.98	.31	3.15	.002	.37	1.59
[Video=Disease]*[Sample=Baseline]	0[a]					
[Video=Control]*[Sample=PV2]	0[a]					
[Video=Control]*[Sample=PV1]	0[a]					
[Video=Control]*[Sample=Baseline]	0[a]					
Video_order	-.14	.21	-.66	.511	-.55	.27
Video_order*[Video=Disease]	.54	.20	2.74	.007	.15	.93
Video_order*[Video=Control]	0[a]					
Video_order*[Sample=PV2]	.25	.15	1.67	.096	-.04	.54
Video_order*[Sample=PV1]	.12	.13	.96	.339	-.13	.37
Video_order*[Sample=Baseline]	0[a]					
Video_order*[Video=D]*[Sample=PV2]	-.71	.21	-3.40	.001	-1.13	-.30
Video_order*[Video=D]*[Sample=PV1]	-.47	.18	-2.63	.009	-.83	-.12
Video_order*[Video=D]*[Sample=Baseline]	0[a]					
Video_order*[Video=C]*[Sample=PV2]	0[a]					
Video_order*[Video=C]*[Sample=PV1]	0[a]					
Video_order*[Video=C]*[Sample=Baseline]	0[a]					

[a]This coefficient is set to zero because it is redundant.

2.4.4 Screenshot disgust rating

After having observed the influence of video order on the spike-specific sIgA secretion we decided to run an additional exploratory analysis on the influence of video order on the screenshot disgust rating, to further understand the nature of the potential interpretational bias. We analyzed the screenshot rating after the control the video as well as the rating after the disease video utilizing a Mann-Whitney-U test and found that participants, who watched the control video first, rated it as significantly more disgusting than participants, that watched the disease video first ($U=3.35$, $p<.001$, , $\eta^2=.71$). This difference, although not significant, was also observed in the rating of the disease video ($U=1.80$, $p=.071$, $\eta^2=.72$, see Fig. S5). Overall, these results suggest, that watching the control video first lead to a higher disgust rating on both days.

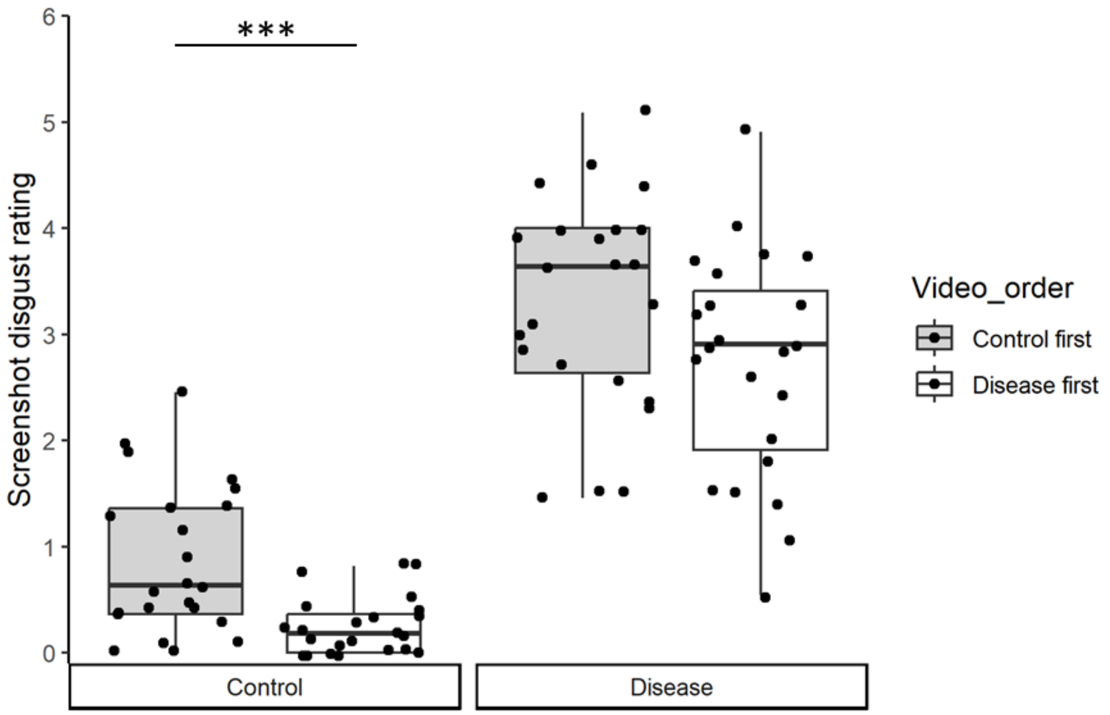

Figure S4: Screenshot disgust rating for the control (left) and disease (right) video of participants that watched the control video first (grey) or disease video first (white). Box represents the median and the 25th and 75th percentiles, whiskers the smallest and the largest value or no further than 1.5*IQR (inter-quartile range). Significant differences are marked with asterisks (***p<.001).

2.5 Difference in interoception scores after the disease video

In order to get a better understanding of the interoception during the disease video we utilized a Wilcoxon-signed-rank test to compare the two composite scores. We found a significant difference between the two interoceptive composite scores related to the disease video. Participants had stronger interoceptive feelings related to the respiratory tract, than gut-related interoceptive feelings ($z=2.78$, $p=.003$, $\eta^2=0.20$) (see Figure S5).

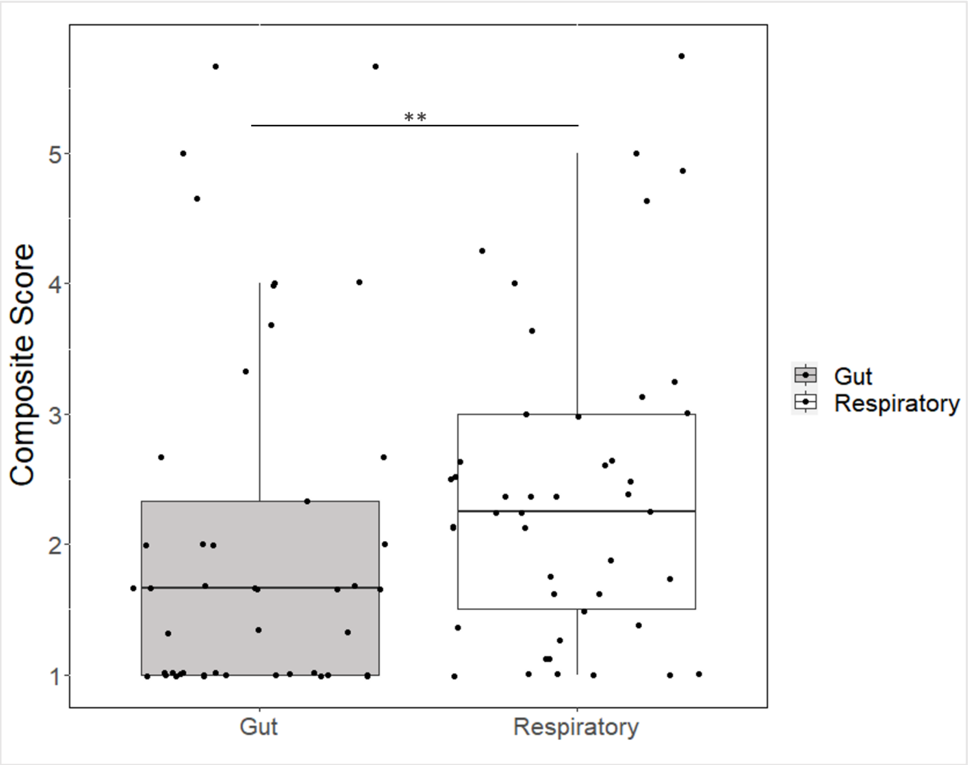

Figure S5: Evaluation of interoceptive feelings after the disease video, regarding the gut (grey) and respiratory (white) composite scores. Box represents the median and the 25th and 75th percentiles, whiskers the smallest and the largest value or no further than 1.5*IQR (inter-quartile range). Significant difference is marked with an asterisk (**p<.01).

2.6 Preregistered GLM-Analysis

Previous to data analysis we preregistered our study with the General Linear Models (GLMs) as primary analysis. After the review process, we decided to switch to GLMM, which may be more appropriate. To keep up transparency of the analyses process, we have added this original analysis here.

2.6.1 SARS-CoV-2-specific sIgA increase after disease-related stimulation

2.6.1.1 Spike-specific sIgA

In order to assess whether the disease video led to an increase in spike-specific sIgA, we performed a 2 x 3 general linear model (GLM) with the factors *Video* (disease, control) and *Sample* (Baseline, Post-Video 1, Post-Video 2). We found a significant main effect of *Video* ($F_{(1,44)}$=4.73, p=.035, ηp^2=.10) and *Sample* ($F_{(2,88)}$=3.33, p=.040, ηp^2=.07), as well as a significant interaction between the two factors ($F_{(2,88)}$=3.71, p=.035, ηp^2=.08). In the post-hoc tests, this was reflected by a significant rise in spike-specific sIgA in the sample directly collected after watching the disease video (Post-Video 1) relative to Baseline (z=-1.80, p=.036, η^2=.72), but not in the corresponding sample taken after the control video (z=-.46, p=.648). Additionally, spike-specific sIgA significantly declined from Post-Video 1 to Post-Video 2 after watching the disease video (z=-2.56, p=.011, η^2=.15), but not after the control video (z=-.12, p=.906). Finally, we found that the samples collected at Post-Video 1 differed significantly between the two videos (disease > control: z=-3.22, p<.001, η^2=.23), while the Baseline (z=-1.59, p=.113) and Post-Video 2 samples (z=-1.25, p=.212) did not (Fig. 1b).

2.6.1.1.1 Exploratory analysis of spike-specific sIgA

Nine of our participants indicated that they were slightly disgusted by the control video. This was unexpected, as the video only displayed healthy people in everyday situations. Since an enhanced feeling of disgust in the control setting could have influenced post-video spike-

specific sIgA, we excluded these participants, who rated more than two of the screenshots from the control video with a score of 4 or higher (this coincided with participants who had an average score above one in the screenshot rating). This left an exploratory sample of 36 participants. In the respective 2 x 3 GLM we found no significant main effect of *Video* ($F_{(1,35)}$=3.81, p=.059), or *Sample* ($F_{(2,70)}$=2.91, p=.061), but replicated the interaction from the total sample ($F_{(2,70)}$=4,34, p=.026, ηp^2=.110).

In the post-hoc tests, we found a significant difference between Baseline and the Post-Video 1 sample after watching the disease video (z=-1.98, p=.024, η^2=.11), but not after watching the control video (z=-.24, p=.648). This was also the case for the difference between the samples taken at Post-Video 1 and Post-Video 2 (Disease: z=-2.75, p=.006, η^2=.21; Control: z=-.58, p=.561). Furthermore, we found that the sample collected at Post-Video 1 differed significantly between the videos (z=-3.39, p<.001, η^2=.32), while the Baseline (z=-1.07, p=.285) and Post-Video 2 sample (z=-1.30, p=.192) did not (see Figure S5a). Finally, the comparison of the rise in spike-specific sIgA from Baseline to Post-Video 1 ($\Delta sIgA_{spike}$) showed that the $\Delta sIgA_{spike}$ of the disease video was significantly higher than the one from the control video (z=-1.95, p=.026, η^2=.11) (see Figure S5b).

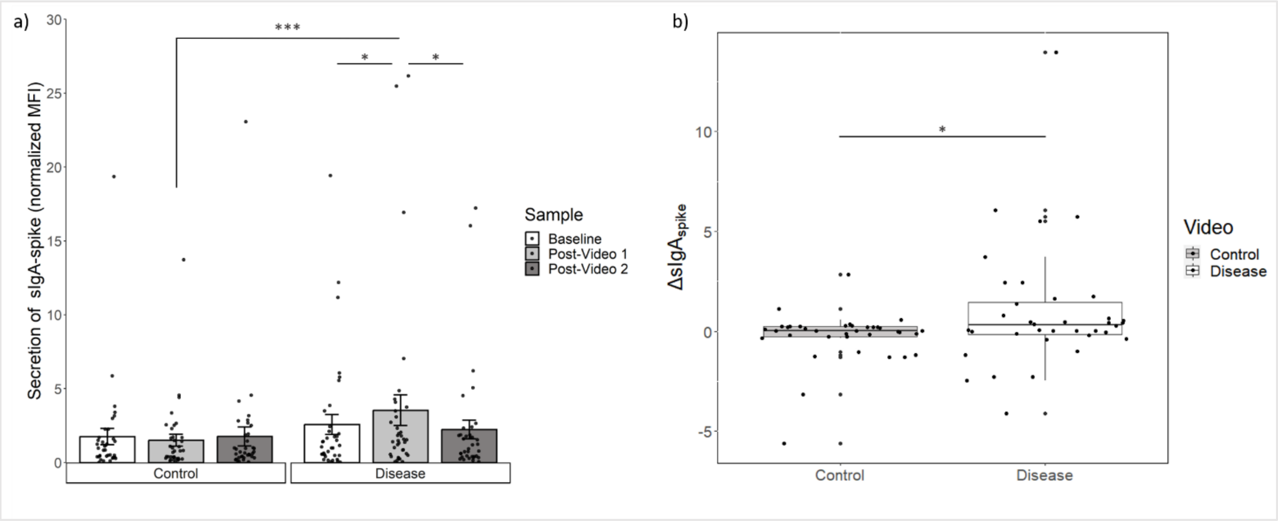

Figure S5: a) Exploratory analysis of spike-specific sIgA in a subgroup of 36 participants. Mean and standard errors of the secretion rate at Baseline, directly after the video (Post-Video 1), and several minutes after the video (Post-Video 2). Significant changes are marked with asterisks (*p<.05; ***p<.001). b) Exploratory analysis of spike-specific ΔsIgA in a subgroup of 36 participants. $\Delta sIgA_{spike}$ of control (grey) and disease (white) video. Box represents the median and the 25th and 75th percentiles, whiskers the smallest and the largest value or no further than 1.5*IQR (inter-quartile range). Significant difference is marked with an asterisk (*p<.05).

2.6.1.1.2 Confirmatory analysis with total sIgA

Finally, we ran a confirmatory analysis of total sIgA, which had significantly increased in response to disease-related video content in our previous study[16]. In the 2 x 3 GLM of total sIgA we found no significant main effect of *Video* ($F_{(1,43)}$=.17, p=.680), but the main effect of *Sample* was significant ($F_{(2,86)}$=10.62, p<.001, ηp^2=.190), as well as the interaction between the factors ($F_{(2,86)}$=6.31, p=.003, ηp^2=.128). In the post-hoc tests, we found a significant difference between the Baseline and the Post-Video 1 sample after watching the disease prime (z=-4.49, p<.001, η^2=.46), as well as after watching the control prime (z=-2.22, p=.027, η^2=.11). However, the increase ($\Delta sIgA_{total}$) after the disease video was significantly higher than the one after the control video (z=-1.75, p=.040, η^2=.07, see Figure S2a). Furthermore, the samples Post-Video 1 and 2 differed significantly after disease video (z=-3.28, p=.001, η^2=.25) but not after the control video (z=-.44, p=.657) (see also Figure S2b).

The $\Delta sIgA_{total}$ was further positively correlated with $\Delta sIgA_{spike}$ for the disease video (rho=.593, p<.001).

2.6.1.2 RBD-specific sIgA

In a second step, we analyzed the RBD-specific sIgA for changes induced by the disease video. In the 2 x 3 GLM we neither found a significant main effect of *Video* ($F_{(1,44)}$=3.03, p=.089, ηp^2=.064) nor of *Sample* ($F_{(2,88)}$=1.12, p=.331, ηp^2=.025), but there was a significant interaction between the two factors ($F_{(2,88)}$=7.60, p<.001, ηp^2=.147). Different from the spike-specific sIgA,

the RBD-specific sIgA showed no significant rise from Baseline to directly after the disease video ($\Delta sIgA_{RBD}$: z=-.37, p=.714), and – similar to the spike-specific sIgA – also not after the control video ($\Delta sIgA_{RBD}$: z=-.04, p=.968). Instead, we found a trend-wise decline in the RBD-specific sIgA from Post-Video 1 to Post-Video 2 (z=-1,95, p=.052, η^2=.08), and also from Baseline to Post-Video 2 (z=-2.18, p=.029, η^2=.11) following the disease video. This indicated a continuous decrease in RBD-specific sIgA throughout the experimental session with the disease video. After the control video, we found a significant increase between Post-Video 1 and Post-Video 2 (z=-2.07, p=.038, η^2=.10), and also when comparing Baseline and Post-Video 2 (z=-2.18, p=.029, η^2=.11) (see Fig. 1c).

3. Supplementary References

Keller, J.K., Wülfing, C., Wahl, J., Diekhof, E.K., 2022. Disease-related disgust promotes antibody release in human saliva. Brain Behav. Immun. - Health 24, 100489. https://doi.org/10.1016/j.bbih.2022.100489

Kupfer, T.R., Fessler, D.M., Wu, B., Hwang, T., Sparks, A.M., Alas, S., Samore, T., Lal, V., Sakhamuru, T.P., Holbrook, C., 2021. The skin crawls, the stomach turns: ectoparasites and pathogens elicit distinct defensive responses in humans. Proc. R. Soc. B 288, 20210376.
